# Supplementary material for: Electron shuttling promotes denitrification and mitigates nitrous oxide emissions in lakes
Source: Nat Commun. 2025 Sep 29;16:8564. doi: 10.1038/s41467-025-63601-0 (PMC12480950; doi:10.1038/s41467-025-63601-0)
Supplement: Supplementary file 1 — Supplementary Information [file 41467_2025_63601_MOESM1_ESM.pdf]

Supplementary Information for

Electron shuttling promotes denitrification and mitigates nitrous oxide emissions in lakes

Kang Song[1,2,3,4,$,*], Yanlin Xiao[1,5,$], Yuren Wang[1,4], Min Deng[1,3,*], Shuni Zhou[1,4], Yongxia Huang[1], Senbati Yeerken[6], Lu Li[1,2], Fengchang Wu[7,*]

[1] State Key Laboratory of Lake and Watershed Science for Water Security, Institute of Hydrobiology, Chinese Academy of Sciences, Wuhan 430072, China

[2] Southern Marine Science and Engineering Guangdong Laboratory (Guangzhou), Guangzhou, 511458, China

[3] National-Regional Joint Engineering Research Center for Soil Pollution Control and Remediation in South China, Guangdong Key Laboratory of Integrated Agro-environmental Pollution Control and Management, Institute of Eco-environmental and Soil Sciences, Guangdong Academy of Sciences, Guangzhou 510650, China

[4] University of Chinese Academy of Sciences, Beijing 100049, China

[5] School of Environmental Studies, China University of Geosciences, Wuhan, Hubei 430074, China

[6] College of Ecology and Environment, Xinjiang University, Urumqi 830046, China

[7] State Key Laboratory of Environmental Criteria and Risk Assessment, Chinese Research Academy of Environmental Sciences, Beijing 100012, China

[$]K.S. and Y.X. contributed equally to this work.

*Corresponding authors.

E-mail addresses: sk@ihb.ac.cn (K.S.), dengmin@ihb.ac.cn (M.D.), wufengchang@vip.skleg.cn (F.W.)

Donghu South road No. 7, Wuhan city, Hubei province, China.

Supplementary Methods

S1. Maximum power density measurement

A microbial fuel cell reactor (Supplementary Fig. S2) was set up to measure the maximum power density (MPD) generated by sediment-based microbial activity. The reactor consisted of two identical chambers: an anode chamber and a cathode chamber. Each chamber had an internal volume of 100 mL and a working volume of 90 mL. Both chambers contained carbon felt electrodes with a surface area of 6 cm^2, separated by a proton exchange membrane (PEM) that was pretreated to enhance performance.

The PEM was treated through a series of steps to improve its conductivity and durability. First, it was soaked in a 5% hydrogen peroxide solution (*w*/*w*) at 80 °C for 1 hour, followed by rinsing in ultrapure water for 30 minutes. Next, the membrane was boiled in a 5% dilute sulfuric acid solution (*w*/*w*) at 80 °C for 1 hour and then boiled again in deionized water at 80 °C for 30 minutes.

In the cathode chamber, 90 mL of a phosphoric acid buffer solution was prepared, containing 19.1 g L^{-1} $Na_2HPO_4 \cdot 12H_2O$ and 2.08 g L^{-1} $NaH_2PO_4 \cdot 2H_2O$. The anode chamber was filled with the same buffer solution, mixed with 10 g of sediment. To ensure homogeneity, the sediment mixture was stirred continuously using a magnetic stirrer at approximately 200 rpm. Air was supplied to the cathode chamber to facilitate dissolved oxygen as the electron acceptor. The anode and cathode electrodes were connected with a titanium wire, and external resistance was applied using a resistance box (ZX21, 0.1 Ω – 99,999.9 Ω).

Voltage (V) was recorded at regular intervals using a multimeter (Model VICTOR 880C). Polarization experiments were conducted across a range of external resistances (100 Ω - 80,000 Ω). Power density (PD), normalized by electrode surface area, was calculated using equation (1):

$$PD = U^2/SR_1 \quad (1)$$

where U denotes the voltage of the microbial fuel cell (mV), R_1 is the external resistances value (100 Ω – 90,000 Ω), and S represents the surface area of the anode and cathode carbon felt (6×10^{-4} m^2). The MPD was obtained from the current density-power density plot (Supplementary Fig. S2).

S2. Dissolved N_2 concentration measurement

Water samples were collected from 30 cm below the surface using a 3 L sampler. To avoid excessive agitation and bubble formation, the collected water was carefully transferred into 12 mL vials through a silicone tube at a controlled flow rate. To further prevent the ingress of atmospheric N_2, the silicone tube was positioned at the bottom of each vial during filling. Next, 200 μL of saturated $ZnCl_2$ solution was added to each vial to inhibit microbial activity. The vials were then sealed and stored at 4°C until further analysis.

The dissolved N_2 concentration in water samples was measured using a membrane inlet mass spectrometer system (HPR-40, Hiden Analytical Co.) following the N_2:Ar method [2]. The system determined N_2:Ar ratios based on the quadrupole instrument signal, which detected the pressures of N_2 and Ar. Finally, the dissolved N_2 concentration was calibrated using water standards equilibrated with air.

S3. Water collection for dissolved N_2O concentration measurement

The dissolved N_2O concentration was measured following a previously reported method [3] with minor modifications. Specifically, water samples for dissolved N_2O analysis were collected 30 cm below the surface using 200 mL plastic syringes fitted with a three-way stopcock. Each syringe was flushed three times with water from the sampling point to avoid contamination, and any air bubbles were carefully expelled before collecting the sample.

Triplicate samples were taken at each location without adding preservatives. The headspace equilibrium method was applied immediately to extract and measure the dissolved N_2O concentration. To achieve this, 40 mL of the water sample in each syringe was displaced with 40 mL of ambient air. Samples for ambient air N_2O measurement were also collected triplicate at each sampling site. The syringes containing the water-air mixture were shaken for 5 minutes to equilibrate the dissolved N_2O in water with the headspace.

Following equilibration, N_2O in the headspace was manually and gently injected into a 12 mL pre-evacuated vial (Labco, UK). The samples were analyzed within 72 hours of collection using a gas chromatograph (7890B Agilent Technologies, Santa Clara, California, USA) [2]. The original concentration of N_2O in water (C_w) prior to equilibrium was calculated using the headspace balancing method, as described in Equation (21):

$$C_w = C_g \times (k_0 \times R \times T + V_g/V_l) - C_{air} \times V_g/V_l \quad (2)$$

where C_g denotes the N_2O concentration in the headspace after equilibrium (μmol L^{-1}), C_{air} is the ambient air N_2O concentration, V_g and V_l are the gas volume (40 mL) and water volume (160 mL) in the vials, respectively, R is the ideal gas constant (0.082 L atm mol^{-1} K^{-1}), T is the absolute temperature during the balancing procedure (K), and k_0 (mol L^{-1} atm^{-1}) is the solubility coefficient of N_2O.

The solubility coefficient of N_2O (k_0) was calculated based on temperature [4] as follows:

$$\ln(k_0) = -62.7062 + 97.3066 \times (100/T) + 24.1406 \times \ln(T/100) \quad (3)$$

where T is Kelvin temperature (K).

S4. Calculation of denitrification and anammox rates

The denitrification rate (D_{total}) and anammox rate (A_{total}) were determined using the $^{15}N\text{-}NO_3^-$ incubation method previously described with some modification [1]. Specifically, the primary nitrogen species in the ^{15}N-labeled incubation bottles were $^{14}N\text{-}NH_4^+$, $^{14}N\text{-}NO_x^-$, and $^{15}N\text{-}NO_x^-$. The initial molar fraction (mol/mol) of ^{15}N in NOx- was calculated using Equation (2):

$$F_N = n(^{15}N\text{- }NO_x^-)/[n(^{15}N\text{- }NO_x^-)+ n(^{14}N\text{- }NO_x^-)] \quad (4)$$

For denitrification (D), assuming that NO_x^- is randomly utilized by denitrifiers, the isotopic composition of N_2 produced during denitrification is as follows:

$$^{14}N\text{-}NO_x^- + {}^{14}N\text{-}NO_x^- \rightarrow {}^{28}N_2 \quad (5)$$

$$^{14}N\text{-}NO_x^- + {}^{15}N\text{-}NO_x^- \rightarrow {}^{29}N_2 \quad (6)$$

$$^{15}N\text{-}NO_x^- + {}^{14}N\text{-}NO_x^- \rightarrow {}^{29}N_2 \quad (7)$$

$$^{15}N\text{-}NO_x^- + {}^{15}N\text{-}NO_x^- \rightarrow {}^{30}N_2 \quad (8)$$

Therefore, the production rates of $^{28}N_2$ (D_{28}), $^{29}N_2$ (D_{29}), and $^{30}N_2$ (D_{30}) from denitrification are:

$D_{28} = D_{total} \times (1\text{-}F_N) \times (1\text{-}F_N)$ (9)

$D_{29} = D_{total} \times (1\text{-}F_N) \times F_N + D_{total} \times F_N \times (1\text{-}F_N)$ (10)

$D_{30} = D_{total} \times F_N \times F_N$ (11)

For anammox (A), the isotopic composition of N_2 produced is:

$^{14}N\text{-}NH_4^+ + {}^{14}N\text{-}NO_x^- \rightarrow {}^{28}N_2$ (12)

$^{14}N\text{-}NH_4^+ + {}^{15}N\text{-}NO_x^- \rightarrow {}^{29}N_2$ (13)

Thus, the production rates of $^{28}N_2$ (A_{28}) and $^{29}N_2$ (A_{29}) from anammox are:

$A_{28} = A_{total} \times (1 - F_N)$ (14)

$A_{29} = A_{total} \times F_N$ (15)

Based on the above derivation, only denitrification can produce $^{30}N_2$. The denitrification rate (D_{total}) can be derived from Equation (9) as:

$D_{total} = D_{30}/(F_N \times F_N)$ (16)

$D_{30} = {}^{30}N_2$ (17)

Therefore,

$D_{total} = {}^{30}N_2/(F_N \times F_N)$ (18)

Further, the anammox rate (A_{total}) can be derived from Equation (13) as:

$A_{total} = A_{29}/F_N$ (19)

$A_{29} = {}^{29}N_2 - D_{29}$ (20)

From Equation (8), we can derive:

$D_{29} = D_{total} \times 2 \times (1\text{-}F_N) \times F_N$ (21)

Combining Equations (15) - (18), we obtain:

$A_{total} = \{{}^{29}N_2 - [{}^{30}N_2 \times 2 \times (1\text{-}F_N)]/F_N\}/F_N$ (22)

Finally, based on $^{30}N_2$ and $^{29}N_2$ production rates (Supplementary Fig. S16-S17) and F_N in the culturing vials, the denitrification rate and anammox rate can be calculated using Equations (16) and (20), respectively.

S5. Functional genes Real-time qPCR

The qPCR standards for *nirS*, *nirK*, *nosZI*, *nosZII*, and 16S rRNA were prepared using environmental DNA extracted from Taihu Lake sediment [8]. Specifically, PCR amplification was performed with premix Taq™ (RR003A, Ex Taq™ Version 2.0, Takara, Bio, Shiga, Japan) with primers listed in Supplementary Table S11. The target DNA was purified using the AxyPrep DNA Gel Extraction Kit (AP-GX-50, Axygen Biosciences, CA, USA) according to the manufacturer's instructions. The purified DNA fragments were cloned into the pMD™ 18-T Vector (6011, Takara Bio, Shiga, Japan) and transformed into *E. coli* JM109 competent cells (9052, Takara Bio, Shiga, Japan). Positive clones were selected on LB plates containing Amp antibiotic, and plasmid DNA was extracted and verified by sequencing.

For extracellular electron transfer (EET) genes (*omcB*, *omcS*, and *pilA*), the full-length gene sequences were obtained from the NCBI database by searching for the whole-genome sequences of model bacteria involved in EET. The primers provided in the references (Supplementary Table S4) and their complementary base pairing were used to obtain the target gene sequences, retaining 10

nucleotides upstream and downstream of the target gene sequences from the whole-genome sequences. The target genes were synthesized and cloned into plasmid bacteria using pUC57 vector (3271, Takara Bio, Shiga, Japan) and One Shot TOP10 Chemically Competent *E. coli* (C404003, Thermo Fisher Scientific, MA, USA) by Wuhan Icongene Gene Technology Co., Ltd. The specific sequences are as follows:

omcB 212 bp
>dbj|AP028967.1|:208725-209026 Geobacter sp. 60473 DNA, complete genome
ATCGCCACGACCCACTTCGACAACTATTCGACCGGTCCCCAGGCCGGTGCCGGCGCTGG
CGGCACCAACGCCAAGGTTGAAGGCTACGTCCTCCGCCGTACCGGCGCCAACCCCTGCT
TCGACTGCCACGGCCACGAGGCGAAGACCAATACCCGTCCGGGTCGTGATGCCACGATC
CACACTGACTGGGCCAAGTCCGCCCACGCCGGTGGCCTGCTGACCGCCAAGTACA

omcS 133 bp
>gb|CP072789.1|:2747491-2747792 Geobacter sulfurreducens strain PL chromosome, complete genome
GGCCGGTACCTGGTTGGCGAAGGCATAGGAGCCGCTCAGGGACTTGGGCTGGTAACCG
GCACCACCAAGGATACGGTATGCACCAACGGCGCCCCAGGCAGTCGGATCGGCGCTGG
TGCTGTAGGAACCGCTGTTCTTGATGGGGAGACCGGT

pilA 116 bp
>dbj|AP028967.1|:1031228-1031378 Geobacter sp. 60473 DNA, complete genome
CGTTGCGATCATCGGTATTCTCGCTGCAATTGCGATTCCGCAGTTCTCGGCGTATCGTGT
CAAGGCGTACAACAGCGCGGCGTCAAGCGACTTGAGAAACCTGAAGACTGCTCTTGAG
TCCGCATTTGCTGATGAT

The concentration of the plasmid DNA was measured using a NanoDrop spectrophotometer (Thermo Fisher Scientific, DE, USA), and copy numbers were calculated based on plasmid size. Standard curves were generated by creating a series of concentration gradients through stepwise dilution of the plasmid DNA.

The qPCR reaction was performed in a 20-μL mixture comprising 10.0 μL Hieff® qPCR SYBR Green Master Mix (11201ES08, Yeasen, Shanghai, China), 0.5 μM of each primer, and 1 μL of DNA template. qPCR assays were conducted in triplicate using a CFX96 Optical Real-Time Detection System (Bio-Rad, Laboratories Inc., Hercules, CA, USA). Annealing temperatures followed those previously described (Supplementary Table S11). PCR conditions were: initial denaturation at 94 °C for 5 min; followed by 40 cycles of denaturation at 94 °C for 30 s, annealing at a specific temperature for 30 s (except for *nosZ* I at 15 s and *nosZ* II at 60 s), extension at 72 °C for 30 s, and final extension at 72 °C for 5 min. All standard curves exhibited an R^2 value greater than 0.98. The amplification efficiency ranged from 90.2% to 110.9%. Melt curve analysis was performed for each gene's amplification products to ensure specificity. Furthermore, the size of each amplification product, including those from the blank extractions and negative controls, was verified by agarose gel electrophoresis to ensure consistency with the expected size (Supplementary Fig. S19).

S6. Library construction, Illumina Miseq sequencing, quality control assembly, amplicon sequence variants (ASVs) clustering, and taxonomy annotation

After genomic DNA extraction from the samples, the conserved region of DNA was amplified using specific primers (341F: 5'-CCTACGGGNGGCWGCAG-3' and 806R: 5'-GACTACHVGGGTATCTAATCC-3') with barcodes. To ensure the accuracy and reliability of the results, blank extraction and negative PCR controls were processed alongside the sediment samples to monitor potential contamination during the extraction and amplification steps. The PCR reaction was set up in a total volume of 50 μL, containing 15 μL of Phusion® High-Fidelity PCR Master Mix (M0531L, New England Biolabs, Ipswich, MA, USA), 0.2 μM primers, 10 ng of genomic DNA template, and nuclease-free water to make up the volume. The PCR was performed under the following conditions: initial denaturation at 98 °C for 1 min, followed by 30 cycles of denaturation at 98 °C for 10 s, annealing at 50 °C for 30 s, and extension at 72 °C for 30 s, with a final extension at 72 °C for 5 min. The resulting PCR products were gel-purified and quantified using a QuantiFluor™ fluorometer. The purified amplicons were then pooled equimolarly to construct the sequencing library using the NEBNext® Ultra™ II DNA Library Prep Kit (E7645XL, New England Biolabs, Ipswich, Massachusetts, USA) according to the manufacturer's instructions. Paired-end sequencing (2×250) were performed on an Illumina Miseq platform by Guangzhou Genedenovo Biotechnology Co., Ltd (Guangzhou, China) following standard protocols. Among all sediment samples, 11 were successfully sequenced, with WY_1 failing to generate a sequencing library.

The raw sequencing reads obtained were processed using the QIIME 2 software package (version QIIME2-2023.5) [3]. First, the paired-end reads were trimmed using Cutadapt (version 2.6) to remove primers, yielding high-quality sequencing data [5]. The "tools import" command was used to import the reads into a qza file in the PairedEndFastqManifestPhred33V2 format. Next, the "demux summarize" command provided a visual assessment of sequence quality, which was evaluated using the interactive tool at https://view.qiime2.org. Except for the 6th base in forward reads and the 213th base in reverse reads, which showed lower quality, the 25th percentile of the quality scores for all bases reached a high value of 37 (Supplementary Fig. S20). This indicates that sequencing quality across most regions of both forward and reverse reads was consistently high, concentrated between 35 and 40, thereby meeting the requirements for subsequent analysis. Based on this high quality, the QIIME 2 plugin "dada2 denoise-paired" was applied with the following parameters: --p-trim-left-f 0 --p-trim-left-r 0 --p-trunc-len-f 0 --p-trunc-len-r 0 [6]. Further details on the analysis are available in the GitHub repository (https://doi.org/10.5281/zenodo.16416644). For taxonomic classification, the SILVA database (silva-138-99-nb-classifier.qza) was trained and applied using the "feature-classifier classify-sklearn" command [7]. Finally, ASV and taxonomy tables were generated using the "tools export" command.

S7. Quantifying the impact of environmental factors on *in situ* denitrification potential

To evaluate the key environmental factors influencing *in situ* denitrification potential (dissolved N_2 concentration and ΔN_2 concentration), we first performed Spearman correlation analysis on all

variables (Supplementary Fig. S4). Variables showing significant correlations with denitrification metrics were then subjected to simple linear regression. However, we observed that aqueous NO_3^--N, NO_2^--N, and NH_4^+-N concentrations exhibited negative correlations with both dissolved N_2 and ΔN_2, contrary to expectations, as dissolved inorganic nitrogen (DIN) typically serves as the substrate for microbial N_2 production. This inconsistency likely arises because sediment DIN (not aqueous DIN) drives N_2 production in lakes [9]. Consequently, aqueous DIN was excluded from subsequent regression analyses.

Next, we assessed residual homoscedasticity and normality for all simple linear regression models using Breusch-Pagan and Shapiro-Wilk tests. Where residuals violated assumptions, variables were log-transformed to meet linear regression requirements. Notably, pH (a logarithmic transformation of H^+) was back-transformed to H^+ concentration for modeling.

Finally, we conducted stepwise multiple linear regression for dissolved N_2 and ΔN_2 using variables meeting residual assumptions. Starting from a null model, bidirectional stepwise selection (forward/backward) via Akaike Information Criterion (AIC) identified optimal predictor combinations (R: MASS package), balancing model fit and complexity.

The optimal model was rigorously validated through:

1. Multicollinearity screening (variance inflation factor (VIF) < 5 threshold).
2. Re-testing residual homoscedasticity (Breusch-Pagan) and normality (Shapiro-Wilk).
3. Significance testing of all retained predictors.

To quantify relative environmental influences, variables were standardized (mean = 0, standard deviation = 1), followed by:

1. Nested model comparisons (ANOVA) to test fit reduction upon variable removal.
2. Ranking predictors by absolute standardized regression coefficients.
3. Visualizing effects via color-bar plots (blue: positive; red: negative; *$p < 0.05$, **$p < 0.01$) (Fig. 1c).

The analysis code is publicly accessible:
GitHub: https://doi.org/10.5281/zenodo.16416644
figshare: https://doi.org/10.6084/m9.figshare.28050473

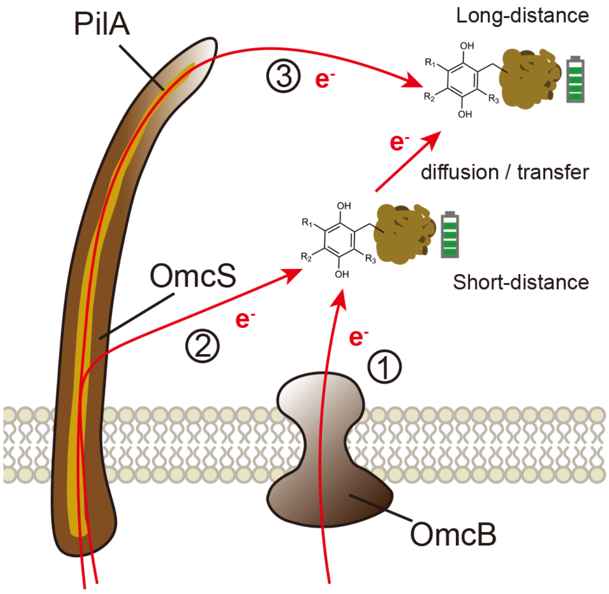

Supplementary Fig. S1 | Schematic diagram of three potential protein-mediated long-distance extracellular electron transfer pathways. Intracellular electrons can be transferred to extracellular electron shuttles, such as humic substances, through three distinct mechanisms: (1) Outer membrane cytochromes (OmcB): Electrons are transferred to extracellular electron shuttles via cytochrome B (OmcB), which is located on the outer membrane of cells. These shuttles then facilitate electron transfer through diffusion or interaction with other electron shuttles, enabling long-distance transfer. (2) Cytochromes on conductive pili (OmcS): Electron are transferred through cytochrome S (OmcS), which is positioned on the surface of extracellular conductive pili. OmcS interacts with electron shuttles to mediate electron transfer over long distances. (3) Physical conductive pathways (PilA): Electrons achieve direct long-distance transfer via a structural protein, PilA, which forms the core of extracellular conductive pili, providing a physical, conductive pathway for electron flow.

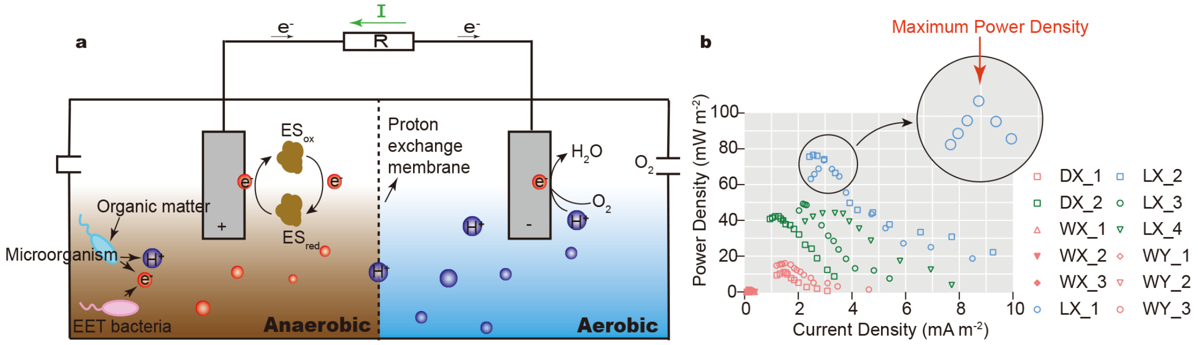

Supplementary Fig. S2 | Measurement of maximum power density of fresh sediments from Lake Taihu. a Schematic diagram of the microbial fuel cell system used to measure the maximum power density (MPD). **b** Current density-power density curve, with the MPD determined from the peak of the plot. Abbreviations: ES, electron shuttles; R, resistance; DX, Daixi river; WX, Wuxi river; LX, Liangxi river; WY, Wangyu river.

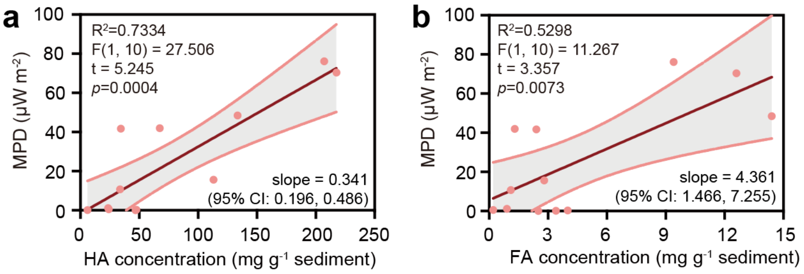

Supplementary Fig. S3 | The relationship between the humic substance concentration and electron transfer capacity. a,b Linear regression analyses depicting the relationships between the concentrations of HA (a) and FA (b) and maximum power density (MPD). Light gray shading with red borders indicates 95% confidence intervals (CI). n = 12 biological replicates. HA, humic acid; FA, fulvic acid.

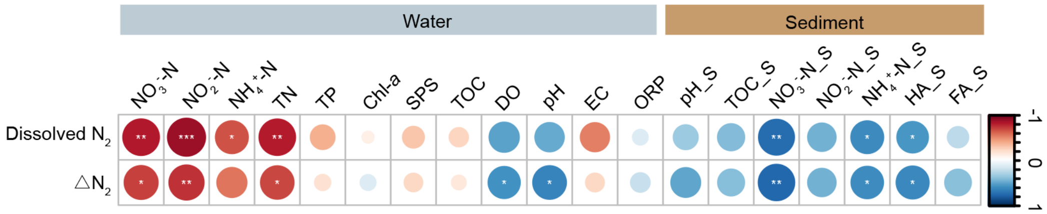

Supplementary Fig. S4 | Spearman correlation between the physicochemical characteristics and the *in situ* emission characteristics of nitrogen gas (N_2). In the correlation matrices, red and blue dots correspond to the negative and positive correlations, respectively. n = 12 biological replicates. Light-colored dots represent low correlations, while dark-colored dots correspond to higher correlations. *, **, and *** represent significantly level at $p < 0.05$, $p < 0.01$, and $p < 0.001$, respectively. Abbreviations: NO_3^--N, nitrate nitrogen; NO_2^--N, nitrite nitrogen; NH_4^+-N, ammonia nitrogen; TN, total nitrogen; TP, total phosphorus; Chl-*a*, chlorophyll *a*; SPS, suspended sediment; TOC, total organic carbon; DO, dissolved oxygen; EC, electrical conductivity; ORP, Oxidation-reduction potential; HA, humic acid; FA, fulvic acid.

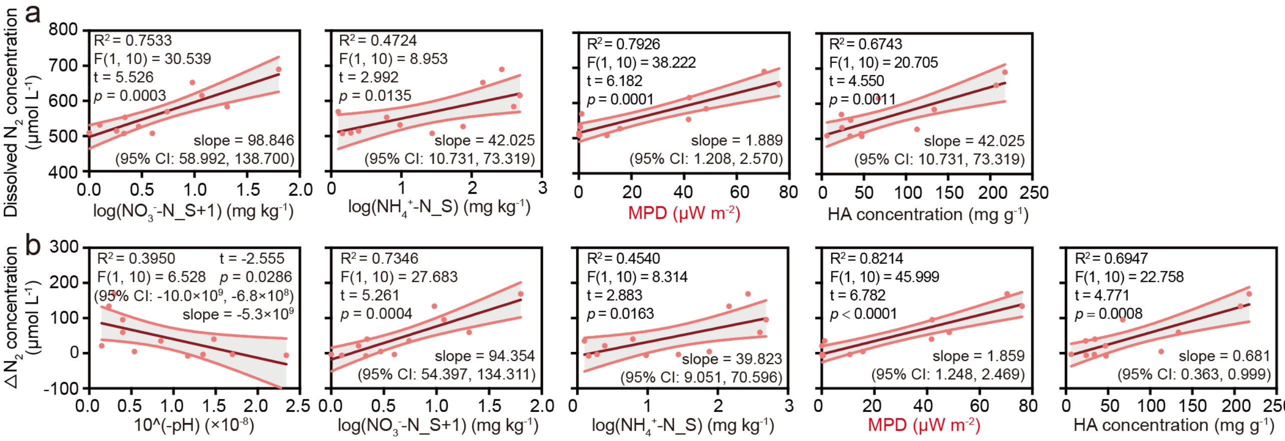

Supplementary Fig. S5 | Linear regression analyses of key environmental factors with N_2 gas emission characteristics. a Linear regression analyses of log(NO_3^--N_S+1), log(NH_4^+-N_S), maximum power density (MPD), and humic acid (HA) concentrations with dissolved N_2 concentration. **b** Linear regression analyses of 10^(-pH) in water, log(NO_3^--N_S+1), log(NH_4^+-N_S), MPD, and HA concentrations with ΔN_2 concentration. n = 12 biological replicates. Light gray shading bordered in red represents the 95% confidence intervals for these regressions. The key physicochemical factors were identified according to the significant level ($p < 0.05$) in Spearman correlation analysis (Supplementary Fig. S3). Only linear regression with a p-value < 0.05 were shown. CI, confidence interval.

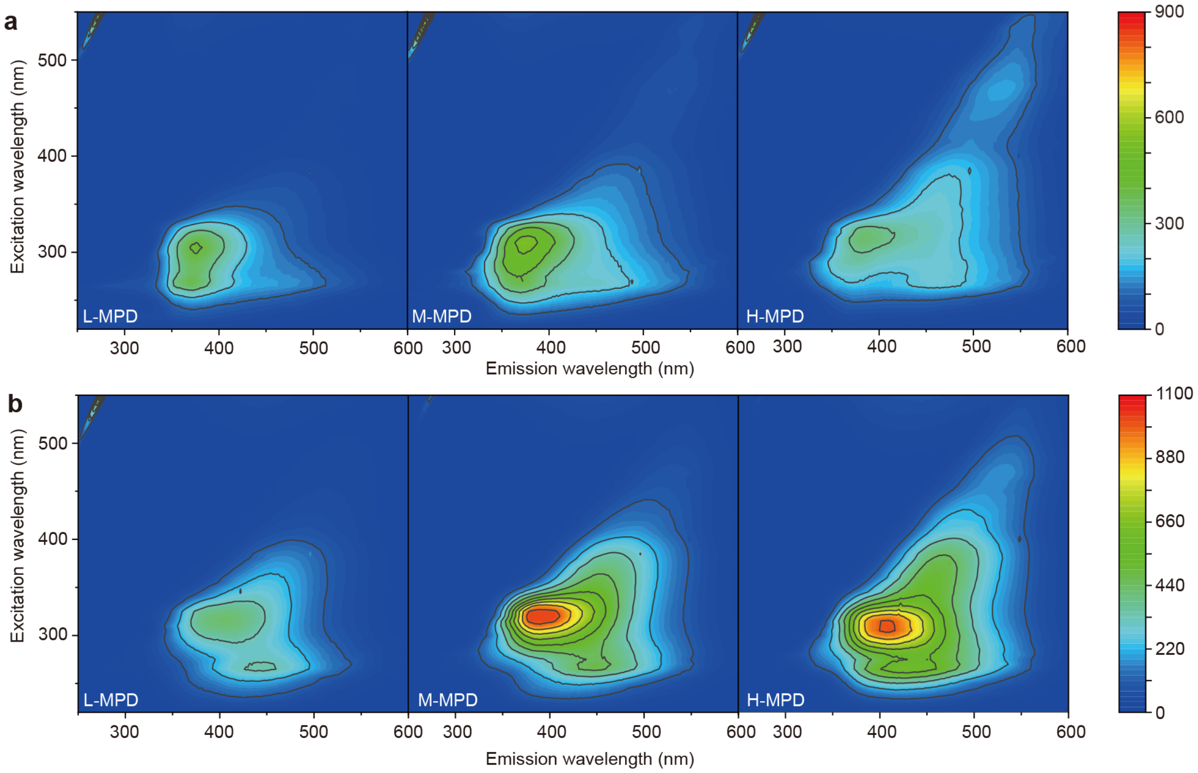

Supplementary Fig. S6 | Excitation-emission matrix (EEM) fluorescence spectroscopy analysis of extracted electron shuttles. a,b EEM fluorescence spectra of humic acid (a) and fulvic acid (b) from representative low-MPD (L-MPD), medium-MPD (M-MPD), and high-MPD (H-MPD) samples. DX_1, LX_3, and LX_1 were randomly selected as representative L-MPD, M-MPD, and H-MPD samples, respectively (Supplementary Table S1). Abbreviation: MPD, maximum power density.

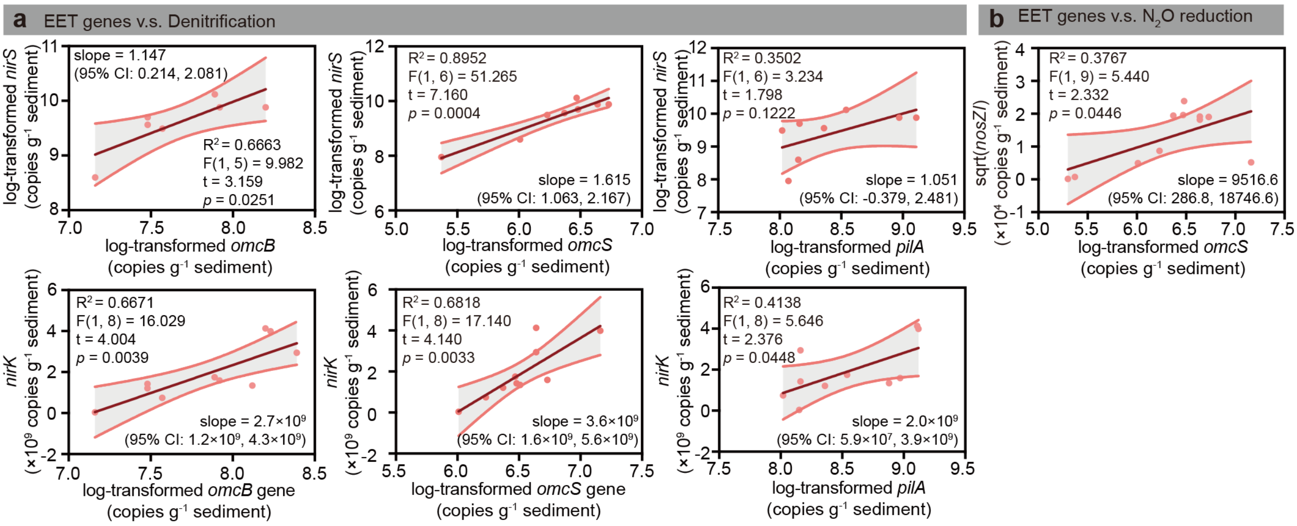

Supplementary Fig. S7 | Simple linear regressions (two-sided) between the absolute abundances of denitrification genes, N_2O reduction gene, and extracellular electron transfer genes. a Linear regression between EET genes (*omcB*, *omcS*, and *pilA*) and denitrifying genes (*nirS* and *nirK*). **b** Linear regression between EET genes and N_2O reducing gene (*nosZI*). Functional genes were log-transformed or sqrt-transformed to meet residual assumptions (homoscedasticity, normality). *n* = 12 biological replicates. The light gray shading with red borders indicates the 95% confidence intervals around the regression lines.

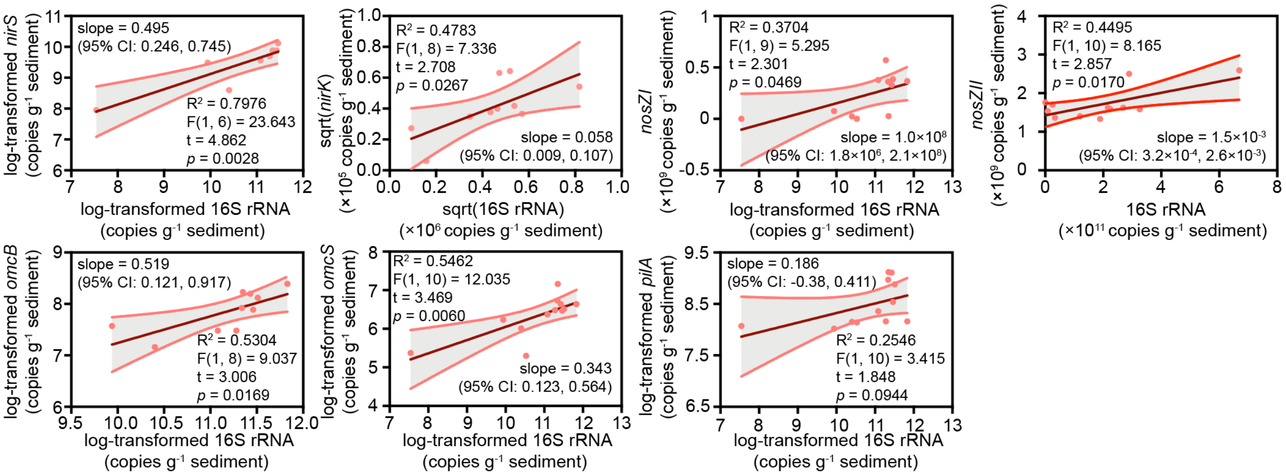

Supplementary Figure S8 | Simple linear regression (two-sided) between denitrification (*nirS* and *nirK*), N_2O reduction (*nosZI* and *nosZII*), extracellular electron transfer (*omcB*, *omcS*, and *pilA*) functional genes and 16S rRNA gene. *n* = 12 biological replicates. Light gray shading with red borders indicates 95% confidence intervals (CI).

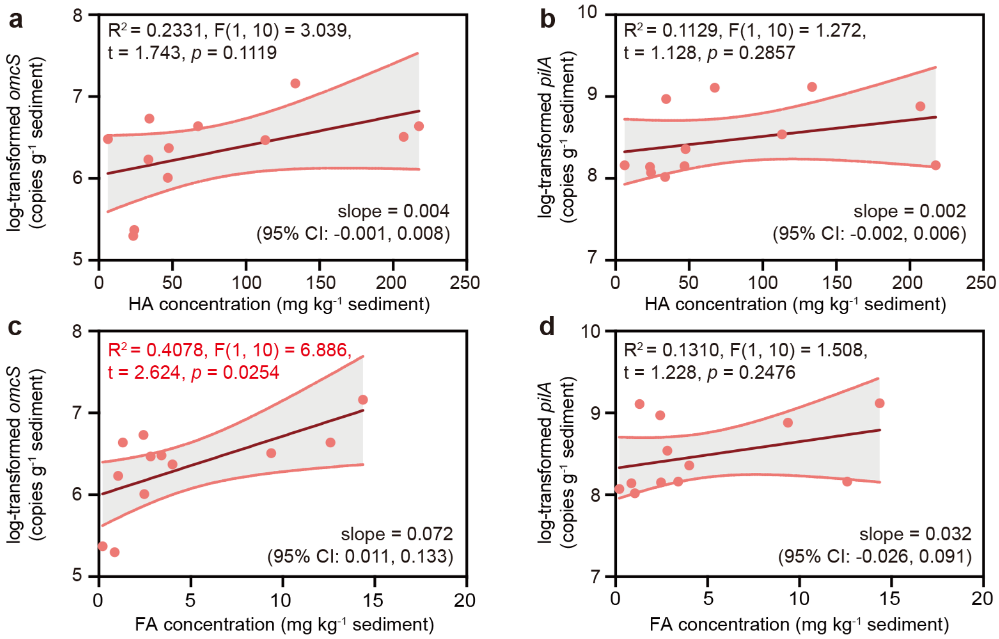

Supplementary Fig. S9 | Relationship between humic substances and extracellular electron transfer (EET) processes. **a** Simple linear regression (two-sided) between humic acid (HA) concentration and *omcS* gene abundance. **b** Simple linear regression (two-sided) between humic acid (HA) concentration and *pilA* gene abundance. **c** Simple linear regression (two-sided) between fulvic acid (FA) concentration and *omcS* gene abundance. **d** Simple linear regression (two-sided) between FA concentration and *pilA* gene abundance. *n* = 12 biological replicates. Light gray shading with red borders indicates 95% confidence intervals.

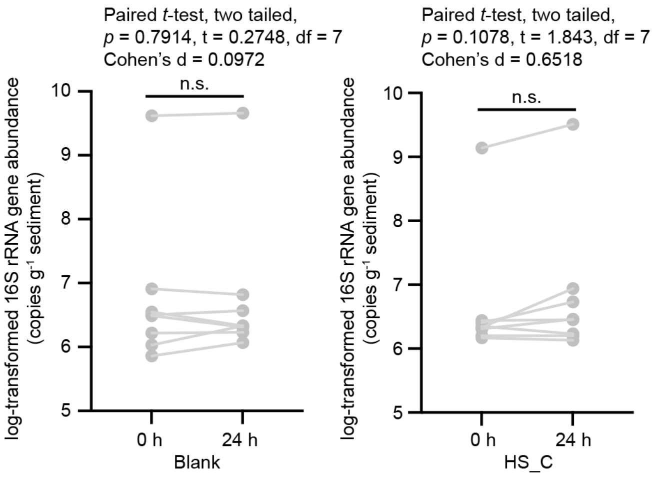

Supplementary Fig. S10 | Bacterial biomass variation during the 24 h incubation period in blank and HS_C group, respectively. *n* = 8 biological replicates. Blank group, no humic substance addition. HS_C group, commercial humic substance addition group.

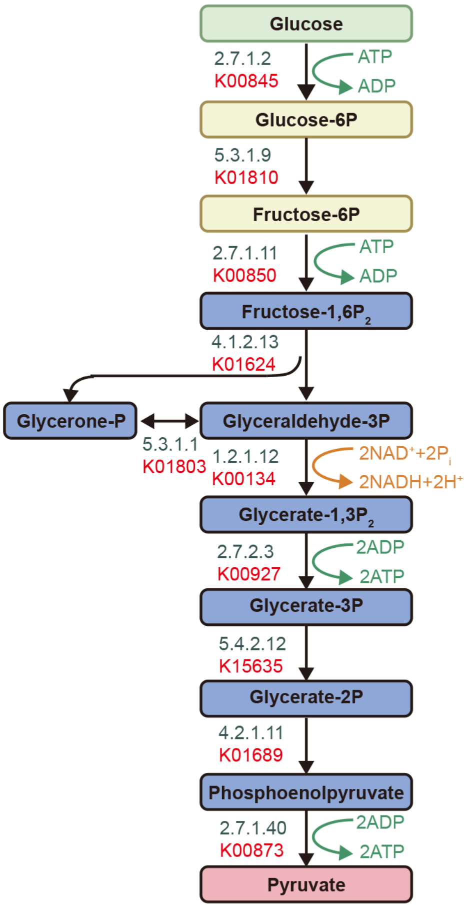

Supplementary Fig. S11 | Genes associated with glycolysis pathways in the recovered MAG LXG.52. Abbreviations: ADP, Adenosine diphosphate; ATP, Adenosine triphosphate.

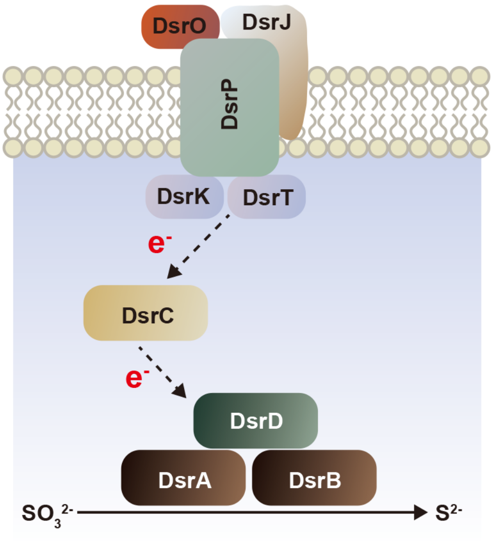

Supplementary Fig. S12 | Genes involved in dissimilatory sulfur reduction pathways in the recovered MAG DX.27.

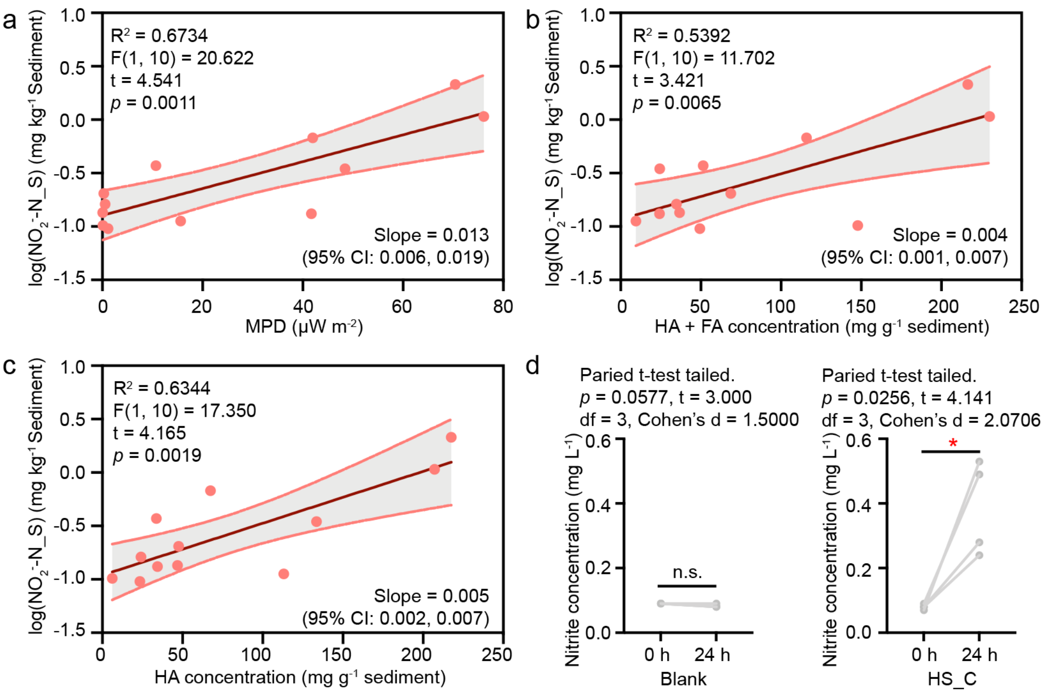

Supplementary Fig. S13 | Effects of maximum power density (MPD) and humic substances on nitrite concentration in sediment. a Simple linear regression (two-sided) between sediment nitrite concentration and MPD. **b** Simple linear regression (two-sided) between sediment nitrite concentration and HA+FA concentration. **c** Simple linear regression (two-sided) between sediment nitrite concentration and HA concentration. *n* = 12 biological replicates for simple linear regression. **d** Temporal variation of nitrite concentration in supernatant during 24-h incubation for Blank (no humic substances addition) and HS_C (commercial humic substances added) groups. *$p < 0.05$. *n* = 3 biological replicates for L-MPD samples (WX_2, WX_3, WY_1).

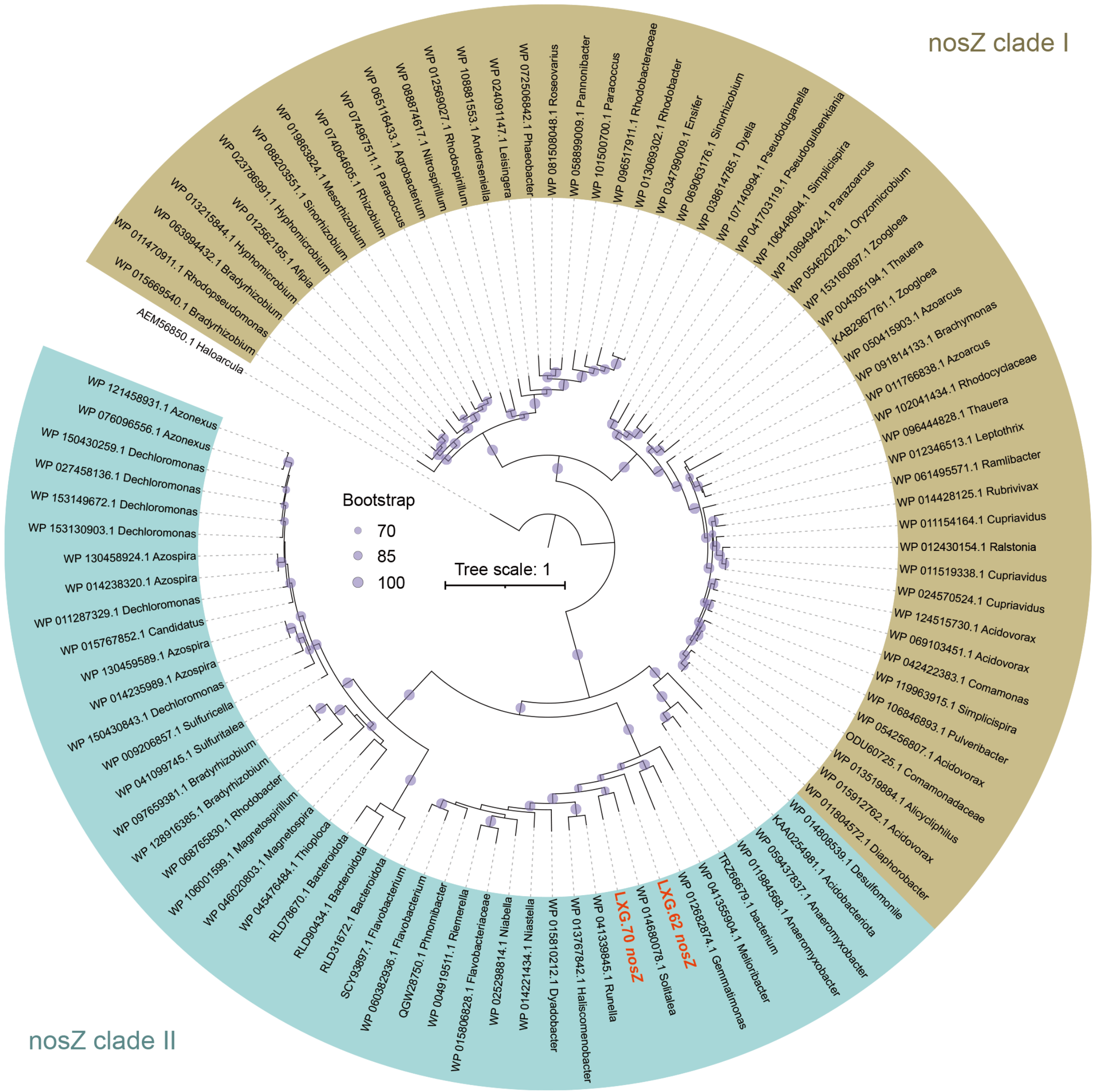

Supplementary Fig. S14 | Maximum-likelihood phylogenetic tree of in silico translated NosZ sequences recovered from the MAGs LXG.62 and LXG.70, and reference NosZ sequences from NCBI's nr database. The best-fit model for NosZ-based phylogenies (based on the large subunits) is LG+F+R8, supported by the Bayesian Information Criterion. The tree was visualized using iTOL v6 (https://itol.embl.de). Leaf colors represent NosZ sequence clusters (nosZ clade I: brown; nosZ clade II: green). Bifurcations with bootstrap values > 70% are marked with open circle symbols to scale.

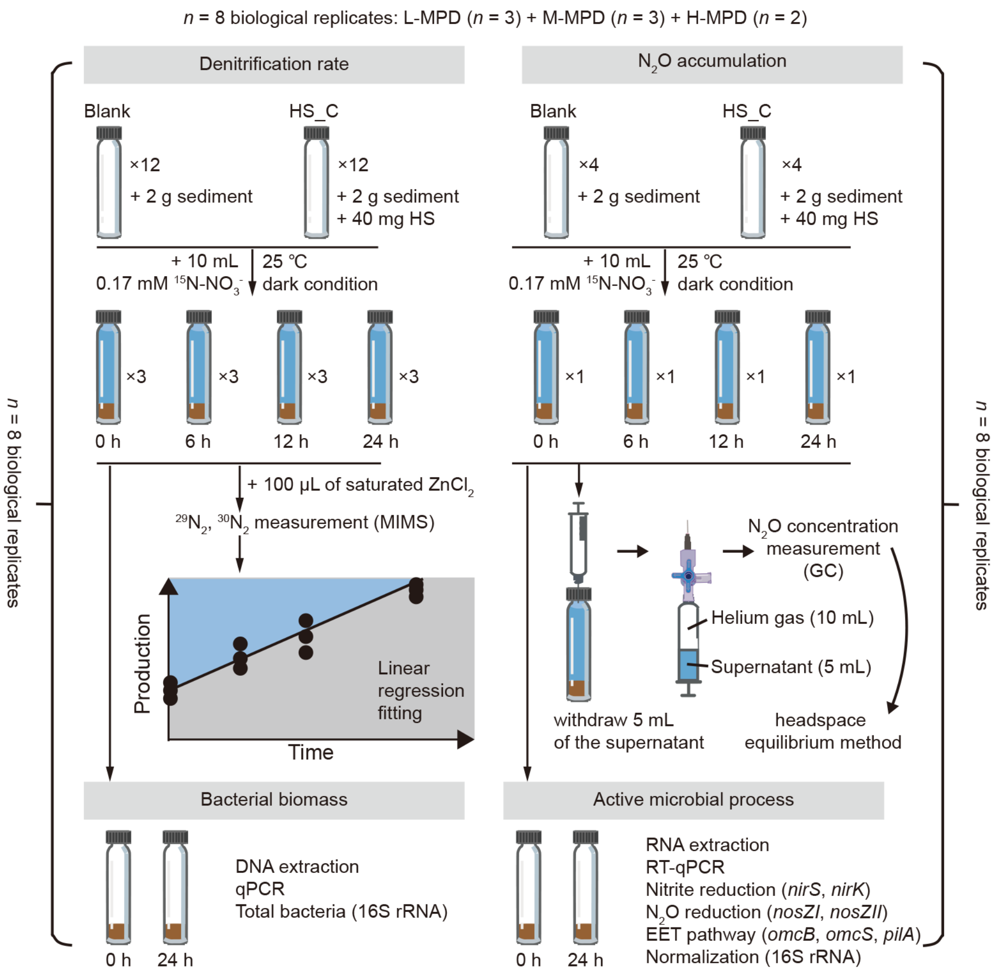

Supplementary Fig. S15 | Workflow for investigating the effects of humic substance addition on denitrification rates, N_2O accumulation concentration, bacterial biomass variation, and active microbial processes. The denitrification rate was determined using ^{15}N isotope tracing method, while N_2O accumulation concentration was measured via the headspace equilibrium method. qPCR was conducted to quantify total bacterial biomass variation during the 24-hour incubation experiment. Reverse transcription quantitative PCR (RT-qPCR) was employed to assess the relative expression levels of three functional gene categories: (1) denitrification genes (*nirS*, *nirK*), (2) N_2O reduction genes (*nosZI*, *nosZII*), and (3) extracellular electron transfer (EET, including *omcB*, *omcS*, and *pilA*) genes. All functional gene expression levels were normalized against the 16S rRNA gene. n = 8 biological replicates, including three L-MPD samples (WX_2, WX_3, and WY_1) and all M-MPD and H-MPD samples (Supplementary Table S1).

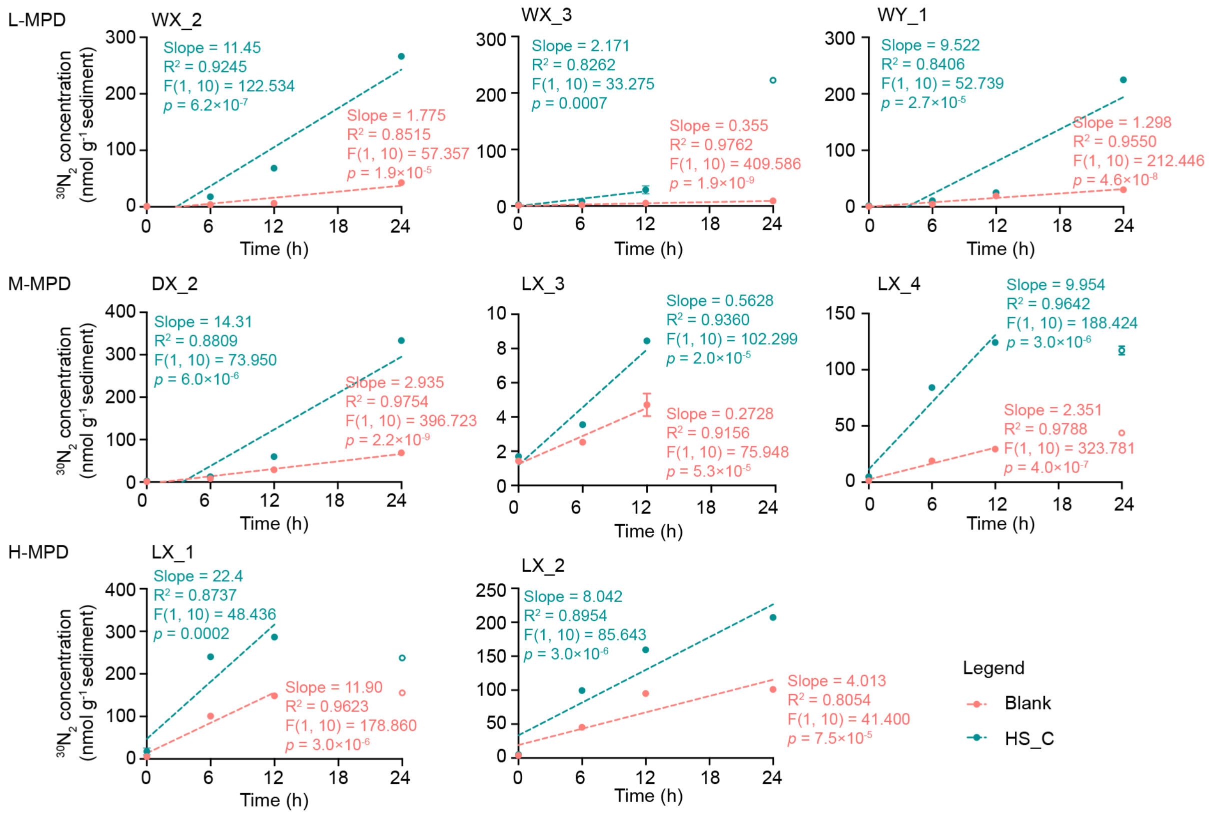

Supplementary Figure S16 | Linear regression analysis of dissolved $^{30}N_2$ concentrations variation (*n* = 8 biological replicates). For LX3, only three time points (0-, 6-, and 12-h) were setup due to insufficient sediment samples. To maximize data retention while ensuring a suitable fitting R^2 ($R^2 > 0.8$), all available data points were included in the regression analysis, excluding the 24-h's data in WX_3, LX_4, and LX_1. Each data point and error bar represent the mean and standard deviation, respectively (*n* = 3 technical replicates). The Blank group denotes sediments without humic substance addition, while HS_C group represents sediments with humic substance addition.

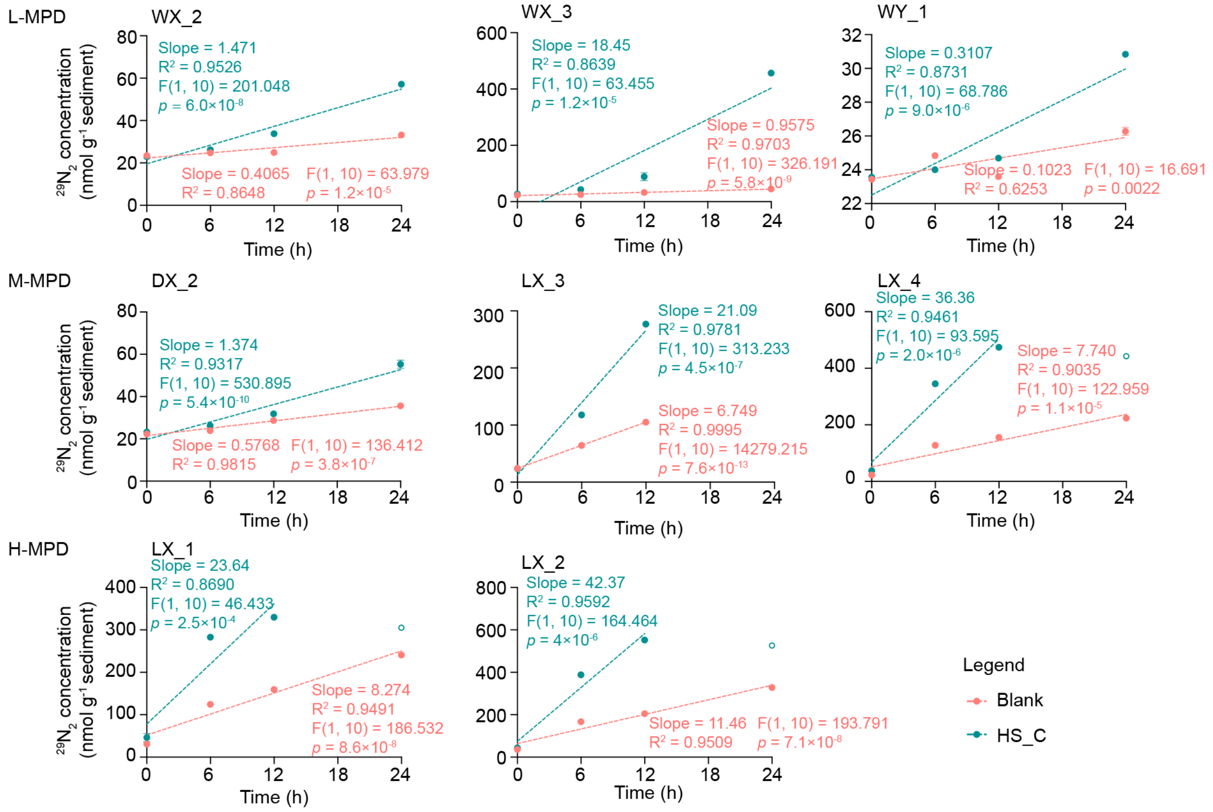

Supplementary Figure S17 | Linear regression analysis of dissolved $^{29}N_2$ concentrations variation (n = 8 biological replicates). For LX3, only three time points (0-, 6-, and 12-h) were setup due to insufficient sediment samples. To maximize data retention while ensuring a suitable fitting R^2 ($R^2 > 0.86$ except for WY_1 ($R^2 = 0.6253$ for Blank group)), all available data points were included in the regression analysis, excluding the 24-h's data in LX_4, LX_1 and LX_2 (). Each data point and error bar represent the mean and standard deviation, respectively (n = 3 technical replicates). The Blank group denotes sediments without humic substance addition, while HS_C group represents sediments with humic substance addition.

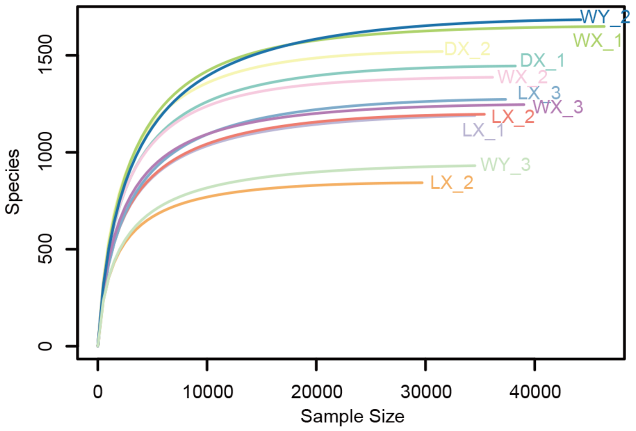

Supplementary Fig. S18 | Rarefaction curve for microbial samples sequenced using Illumina Miseq. Abbreviations: DX, Daixi river; WX, Wuxi river; LX, Liangxi river; WY, Wangyu river.

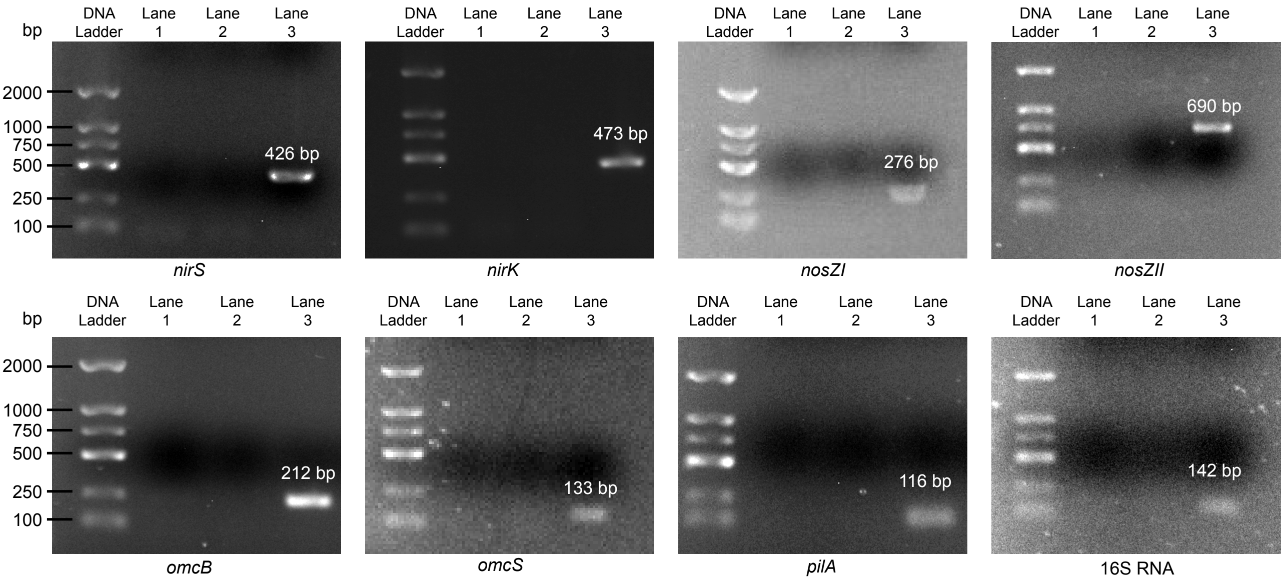

Supplementary Fig. S19 | Agarose gel electrophoresis analysis of qPCR amplicons. Lanes 1, 2, and 3 represent the negative control (Sterile water used in place of nucleic acid sample), the blank extraction (no sediment added during nucleic acid extraction), and the experimental sample, respectively. All gel electrophoresis assays were independently repeated at least twice, yielding consistent results. The raw, uncropped gel images supporting these findings have been deposited in figshare (10.6084/m9.figshare.29856992).

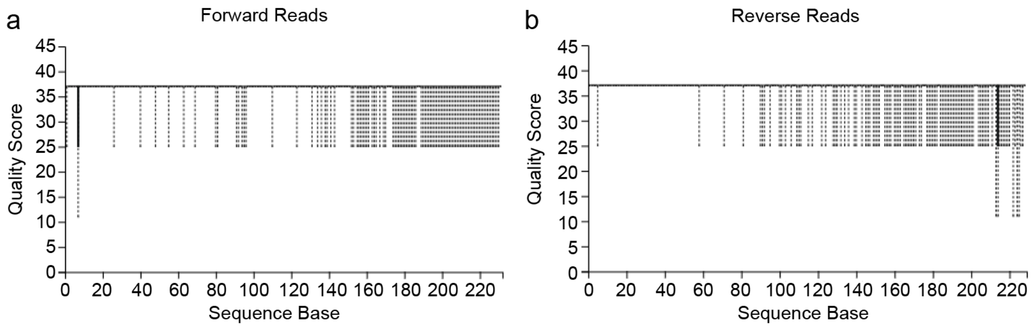

Supplementary Fig. S20. Quality Plot of forward reads and reverse reads. a The quality score of sequence base in forward reads. **b** The quality score of sequence base in reverse reads. Box plots were generated using a random sampling of 10,000 sequences out of 1,221,321, without replacement (*n* = 11 biological replicates). Except for the 6th base in forward reads and the 213th base in reverse reads, the 25th percentile of the quality scores for all bases is as high as 37. Given this high quality, all bases were retained for subsequent analyses.

Supplementary Table S1 | Maximum power density (MPD) levels and dissolved humic substance concentrations of sediment in lake Taihu.

MPD Levels	Sampling sites	MPD ($\mu W\ m^{-2}$)	HA concentration ($mg\ g^{-1}$ sediment)	FA concentration ($mg\ g^{-1}$ sediment)
L-MPD	WX_1	0.06±0.01	46.87	2.47
	WX_2	0.09±0.03	6.12	3.41
	WX_3	0.29±0.02	47.52	4.01
	WY_1	0.56±0.01	24.10	0.21
	WY_2	1.12±0.02	23.46	0.86
	WY_3	15.63±0.40	113.07	2.82
	DX_1	10.70±0.49	33.74	1.05
M-MPD	DX_2	41.69±0.63	34.43	2.43
	LX_3	48.44±1.12	133.52	14.36
	LX_4	41.97±2.21	67.44	1.30
H-MPD	LX_1	70.40±2.77	217.53	12.59
	LX_2	76.13±0.53	207.11	9.37

Abbreviations: L-MPD, Low-MPD; M-MPD, medium-MPD; H-MPD, high-MPD; HA, humic acid; FA, fluvic acid; WX, Wuxi river; WY, Wangyu river; DX, Daixi river; LX, liangxi river.

Supplementary Table S2 | Physicochemical characteristics of overlying water and sediment in Taihu lake.

	Parameters	Minimum	Maximum	Mean±SD	Z score
Water	Temperature (°C)	21.3±0.5	21.5±1.6	21.1±0.3	-2.25~0.75
	pH	7.3	8.9	8.2 ± 0.4	-1.43~1.70
	ORP (mV)	-41.0	29.3	-1.9±20.6	-1.81~1.44
	EC (µS cm^{-1})	437.0	502.0	475.6 ± 20.5	-1.53~1.13
	SPS ((mg L^{-1})	55.0	191.0	102.3±29.3	-1.11~2.27
	Wind speed (m S^{-1})	1.2±0.5	1.5±0.2	1.2±0.8	-1.60~1.54
	DO (mg L^{-1})	6.0	13.6	8.4 ± 2.0	-1.19~2.51
	TOC (mg L^{-1})	23.3	36.8	30.8±5.1	-1.41~1.13
	TP (mg L^{-1})	0.05	0.26	0.13±0.05	-1.46~2.09
	Chl-a (µg L^{-1})	6.8	61.5	22.4±13.8	-1.10~2.37
	NO_3^--N (mg L^{-1})	0.2	2.1	0.9±0.7	-1.02~1.66
	NO_2^--N (mg L^{-1})	0.02	0.10	0.04±0.03	-0.77~1.85
	NH_4^+-N (mg L^{-1})	0.1	1.2	0.5±0.3	-0.98~1.92
	TN (mg L^{-1})	0.3	3.0	1.3±0.9	-0.95~1.68
Sediment	pH	6.6	7.8	7.3±0.4	-1.83~1.51
	MPD (mW m^{-2})	0.1	76.2	26.3±29.4	-0.88~1.76
	TOC (g kg^{-1})	0.09	3.27	0.91 ± 0.91	-0.91~2.60
	NO_3^--N (mg kg^{-1})	0.0	61.5	9.4±17.4	-0.54~2.99
	NO_2^--N (mg kg^{-1})	0.1	2.1	0.5 ± 0.6	-0.61~2.77
	NH_4^+-N (mg kg^{-1})	1.3	490.7	119.0 ± 173.0	-0.68~2.14

Results are presented as mean ± standard deviation (SD) (n = 12 biological replicates). Abbreviations: ORP, oxidation-reduction potential; EC, electrical conductivity; SPS, suspended sediment; DO, dissolved oxygen; TOC, total organic carbon; TP, total phosphorus; Chl-*a*, Chlorophyll *a*; TN, total nitrogen; MPD, maximum power density.

Supplementary Table S3 | Coefficients of the stepwise multiple linear regression model for dissolved N_2 concentration.

Variable	Estimate	t value	Pr(>|t|)
MPD	1.16	2.745	0.0227*
$\log(NO_3^-\_S+1)$	49.54	2.183	0.0569

Adjusted $R^2 = 0.8343$, $p < 0.0002$. Homogeneity of variance (studentized Breusch-Pagan (BP) test): BP = 2.9972, $p = 0.2234$. Normality of residuals (Shapiro-Wilk normality test): W = 0.9422, $p = 0.5264$. $^*p < 0.05$ ($p = 0.0227$).

Supplementary Table S4 | Coefficients of the stepwise multiple linear regression model for ΔN_2 concentration.

Variable	Estimate	t value	Pr(>|t|)
MPD	1.66	4.546	0.0039**
DO	10.28	2.090	0.0816
log(NO_3^-_S+1)	49.34	2.364	0.0560
10^(- pH)	2.17×10^9	1.199	0.2756
log(NH_4^+)	-10.81	-1.060	0.3299

Adjusted $R^2 = 0.8978$, $p < 0.0011$. Homogeneity of variance (studentized Breusch-Pagan (BP) test): BP = 4.7903, $p = 0.4420$. Normality of residuals (Shapiro-Wilk normality test): W = 0.9032, $p = 0.1746$.
** $p < 0.01$ ($p = 0.0039$).

Supplementary Table S5 | Comparison of prediction model goodness-of-fit for dissolved N_2 concentration using ANOVA.

Model	Res.Df	RSS	Df	Sum of Sq	F	Pr(>F)
1	10	2.2811				
2	9	1.4914	1	0.7897	4.7655	0.0569

Model 1: scale(dissolved N_2) ~ scale(MPD); Model 2: scale(dissolved N_2) ~ scale(MPD) + scale(log(NO_3^-_S+1)). Environmental factors and ΔN_2 concentrations were standardized (mean = 0, standard deviation = 1) using the scale function in R software. The two-sided ANOVA comparison indicated no significant improvement in goodness-of-fit for Model 2 versus Model 1 (Pr(>F) > 0.05). Res.Df, Residual degrees of freedom; RSS, Residual sum of squares; Df, Degrees of freedom; Sum of Sq, Sum of squares; Pr(>F), Probability of F-statistic.

Supplementary Table S6 | Comparison of prediction model goodness-of-fit for ΔN_2 concentration using ANOVA.

Model	Res.Df	RSS	Df	Sum of Sq	F	Pr(>F)
1	10	1.9643				
2	6	0.6134	4	1.3509	3.3035	0.0933

Model 1: scale(ΔN_2) ~ scale(MPD); Model 2: scale(ΔN_2) ~ scale(MPD) + scale(DO) + scale(log(NO_3^-_S+1)) + scale(10^(-pH)) + scale(log(NH_4^+)). Environmental factors and ΔN_2 concentrations were standardized (mean = 0, standard deviation = 1) using the scale function in R software. The two-sided ANOVA comparison indicated no significant improvement in goodness-of-fit for Model 2 versus Model 1 (Pr(>F) > 0.05). Res.Df, Residual degrees of freedom; RSS, Residual sum of squares; Df, Degrees of freedom; Sum of Sq, Sum of squares; Pr(>F), Probability of F-statistic.

Supplementary Table S7 | In situ denitrification potential across sampling sites categorized by maximum power density (MPD).

Groups	MPD (μW m^{-2})	Dissolved N_2 concentration (μmol L^{-1})	ΔN_2 concentration (μmol L^{-1})
L-MPD	4.1±6.1	524.0±22.3	5.9±16.2
M-MPD	44.0±3.5	584.2±30.9	65.0±28.1
H-MPD	73.3±3.6	671.1±26.3	151.3±24.6

The detailed sample names for each group are provided in Supplementary Table S1. Abbreviations: L-MPD, Low-MPD; M-MPD, medium-MPD; H-MPD, high-MPD; ΔN_2, excess dissolved N_2.

Supplementary Table S8 | Relative expression levels of denitrifying genes and N_2O reduction genes, and extracellular electron transfer (EET) genes at 0 h.

Samples	Processes	Genes	Blank	HS_C	
All	Denitrifying	*nirS* ($\times 10^{-2}$)	2.5 (1.0)	3.9 (1.8)	p = 0.1656 [a]
	genes	*nirK* ($\times 10^{-3}$)	5.6 (3.0)	1.4 (0.3)	p = 0.2500 [b]
	N_2O-reducing	*nosZI* ($\times 10^{-4}$)	4.6 (1.0)	6.3 (1.9)	p = 0.2736 [a]
	genes	*nosZII* ($\times 10^{-3}$)	9.6 (5.4)	1.2 (0.4)	p = 0.1484 [b]
	EET genes	*omcB* ($\times 10^{-5}$)	2.8 (1.2)	0.6 (0.3)	p = 0.1177 [a]
		omcS ($\times 10^{-5}$)	3.9 (2.2)	1.1 (0.4)	p = 0.3828 [b]
		pilA ($\times 10^{-5}$)	9.7 (5.3)	3.7 (1.3)	p = 0.7422 [b]
L-MPD	Denitrifying	*nirS* ($\times 10^{-4}$)	3.5 (1.8)	6.4 (2.3)	p = 0.2500 [b]
	genes	*nirK* ($\times 10^{-3}$)	1.6 (0.1)	2.0 (0.6)	p = 0.7500 [b]
	N_2O-reducing	*nosZI* ($\times 10^{-4}$)	2.1 (0.5)	4.1 (1.6)	p = 0.2836 [a]
	genes	*nosZII* ($\times 10^{-3}$)	2.4 (0.8)	1.9 (0.6)	p = 0.7436 [a]
	EET genes	*omcB* ($\times 10^{-5}$)	0.9 (0.4)	1.1 (0.7)	p = 0.7814 [a]
		omcS ($\times 10^{-5}$)	1.5 (0.8)	2.1 (0.7)	p = 0.4476 [a]
		pilA ($\times 10^{-5}$)	3.1 (1.2)	6.9 (2.4)	p = 0.2073 [a]

Values were presented as mean (standard error of mean). All functional gene expression levels were normalized to the 16S rRNA gene. n = 8 biological replicates for All samples and n = 3 biological replicates for L-MPD samples. The Shapiro-Wilk test was used to examine the normality of the residuals of the paired samples. If the data met the normality assumptions, a two-sided paired t-test was applied; otherwise, the two-sided Wilcoxon matched-pairs signed rank test was used (GraphPad Prism, version 9.0.0). [a] Paired t-test was used; [b] Wilcoxon matched-pairs signed rank test was used.

Supplementary Table S9 | Relative expression levels of denitrifying genes and N_2O reduction genes, and extracellular electron transfer (EET) genes at 24 h.

Processes	Genes	Blank	HS_C	HS_C/Blank
Denitrifying	*nirS*	0.2685 (0.1043)	1.4880 (1.0480)	4.6 ± 1.6
genes	*nirK*	0.0383 (0.0243)	0.1991 (0.0986)	98.9 ± 63.0
N_2O-reducing	*nosZI*	0.0035 (0.0020)	0.0164 (0.0069)	33.2 ± 15.8
genes	*nosZII*	0.0670 (0.0416)	0.3388 (0.1555)	106.4 ± 68.8
EET genes	*omcB*	0.0002 (0.0001)	0.0012 (0.0005)	74.2 ± 32.2
	omcS	0.0006 (0.0004)	0.0057 (0.0025)	396.1 ± 274.4
	pilA	0.0001 (0.0001)	0.0019 (0.0009)	201.8 ± 115.3

Values were presented as mean (standard error of mean) (n = 8 biological replicates). All functional gene expression levels were normalized to the 16S rRNA gene.

Supplementary Table S10. Geographic information of sampling sites.

Rivers	Sites	Longitude	Latitude
Wuxi	WX_1	120°0′34″	31°27′11″
	WX_2	120°0′32″	31°27′11″
	WX_3	120°0′31″	31°27′12″
Wangyu	WY_1	120°24′17″	31°26′38″
	WY_2	120°24′14″	31°26′36″
	WY_3	120°24′12″	31°26′34″
Daixi	DX_1	120°2′24″	31°29′5″
	DX_2	120°2′22″	31°29′6″
	LX_1	120°13′57″	31°33′4″
	LX_2	120°13′47″	31°33′0″
	LX_3	120°13′60″	31°33′7″
	LX_4	120°14′5″	31°33′66″

Supplementary Table S11 | Primers used for Real-time PCR (qPCR) amplification in this study.

Target genes	Primers	Sequence (5'-3')	Gene size (bp)	Annealing temperature (℃)	Supplementary References
omcB	8912	CCCACTTCGACAACTATTCG	212	58	[10]
	8908-2	GGTCAGCAGGCCACCGG			
omcS	omcS-F	CCATCAAGAACAGCGGTTCC	133	60	[11]
	omcS-R	TGGTTGGCGAAGGCATAGG			
pilA	pilA-F	ATCGGTATTCTCGCTGCAAT	116	60	[11]
	pilA-R	AATGCGGACTCAAGAGCAGT			
nirS	cd3aF	GTSAACGTSAAGGARACSGG	426	57	[12]
	R3cd	GASTTCGGRTGSGTCTTGA			
nirK	F1aCu	ATCATGGTSCTGCCGCG	473	55	[13]
	R3Cu	GCCTCGATCAGRTTGTGGTT			
nosZI	nosZ2F	CGCRACGGCAASAAGGTSMSSGT	276	60	[14]
	nosZ2R	CAKRTGCAKSGCRTGGCAGAA			
nosZII	nosZ-II-F	CTIGGICCIYTKCAYAC	690	54	[15]
	nosZ-II-R	GCIGARCARAAITCBGTRC			
Bacterial 16S rRNA	16SrRNA-F	CGGTGAATACGTTCYCGG	142	55	[16]
	16SrRNA-R	GGHTACCTTGTTACGACTT			

Supplementary Table S12 | The α-diversity indices of microbial communities in sediment samples.

MPD levels	Sampling sites	ASVs	Chao1	Shannon	Simpson	Goods coverage
L-MPD	WX_1	1582	1618.0	6.48	0.996	0.997
	WX_2	1331	1355.3	6.40	0.997	0.998
	WX_3	1196	1231.0	6.16	0.995	0.997
	WY_2	1587	1657.5	6.39	0.995	0.995
	WY_3	879	902.0	5.49	0.986	0.998
	DX_1	1380	1412.6	6.33	0.995	0.997
M-MPD	DX_2	1452	1488.3	6.58	0.997	0.997
	LX_3	1083	1123.1	6.03	0.994	0.997
	LX_4	1111	1136.2	6.04	0.994	0.998
H-MPD	LX_1	791	799.7	5.54	0.989	0.999
	LX_2	1167	1202.2	6.19	0.996	0.998

Supplementary Table S13. Statistical analysis information.

		Normality of Residuals	Paired *t*-test			Wilcoxon matched-pairs signed rank test
		Shapiro-Wilk (p value)	t	df	p value (two-tailed)	p value (two-tailed)
MPD	All	0.4848	0.9046	7	0.3957	-
	L-MPD	0.5246	9.163	2	0.0117	-
D_{total}	All	0.6130	4.510	7	0.0028	-
C_{N2O}	All	0.0105	-	-	-	0.0156
nirS/16S rRNA	All	0.0770	3.341	7	0.0124	-
nirK/16S	All	0.2548	0.6325	7	0.5472	-
rRNA	L-MPD	0.2025	7.111	2	0.0192	-
nosZI/16S	All	0.1428	0.8431	7	0.4270	-
rRNA	L-MPD	0.0675	23.70	2	0.0018	-
nosZII/16S	All	0.1865	0.5225	7	0.6174	-
rRNA	L-MPD	0.0893	6.944	2	0.0201	-
omcB/16S	All	0.0634	0.9950	7	0.3529	-
rRAN	L-MPD	0.5600	11.14	2	0.0080	-
omcS/16S	All	0.4857	0.7625	7	0.4707	-
rRNA	L-MPD	0.4291	4.874	2	0.0396	-
pilA/16S	All	0.2729	0.9055	7	0.3953	-
rRNA	L-MPD	0.8322	6.843	2	0.0207	-

MPD, maximum power density; D_{total}, Denitrification rate; C_{N2O}, Dissolved N_2O concentration; All, All groups (n = 8) included three biological replicates in Low-MPD (L-MPD) group, three biological replicates in medium-MPD and two biological replicates in high-MPD groups. L-MPD, low-MPD group (WX_2, WX_3, and WY_1). Normality of residuals between paired samples (blank and HS-added) was assessed using the Shapiro-Wilk test. For metrics with normal residuals ($p > 0.05$), a paired *t*-test was used, while the Wilcoxon matched-pairs signed rank test was applied for non-normal residuals ($p < 0.05$). All analyses were performed using GraphPad Prism (Version 9.0.0).

Supplementary References

1. Thamdrup B, Dalsgaard T. Production of N_2 through anaerobic ammonium oxidation coupled to nitrate reduction in marine sediments. *Appl. Environ. Microb.* **68**, 1312-1318 (2002).
2. Zhou Y*, et al.* Nonlinear pattern and algal dual-impact in N_2O emission with increasing trophic levels in shallow lakes. *Water Res.* **203**, 117489 (2021).
3. Bolyen E, et al. Interactive, scalable and extensible microbiome data science using QIIME 2. *Nat. Biotechnol.* **37**, 852-857 (2019).
4. Wanninkhof R. Relationship between wind speed and gas exchange over the ocean revisited. *Limnol. Oceanogr-Meth.* **12**, 351-362 (2014).
5. Martin M. Cutadapt removes adapter sequences from high-throughput sequencing reads. EMBnet.journal **17**, 10-12 (2011).

6. Callahan BJ, McMurdie PJ, Rosen MJ, Han AW, Johnson AJA, Holmes SP. DADA2: High-resolution sample inference from Illumina amplicon data. *Nat. Methods* **13**, 581-583 (2016).
7. Mosley OE, Gios E, Close M, Weaver L, Daughney C, Handley KM. Nitrogen cycling and microbial cooperation in the terrestrial subsurface. *ISME J.* **16**, 2561-2573 (2022).
8. Song K, Senbati Y, Li L, Zhao X, Xue Y, Deng M. Distinctive microbial processes and controlling factors related to indirect N_2O emission from agricultural and urban rivers in Taihu watershed. *Environ. Sci. Technol.* **56**, 4642-4654 (2022).
9. Zhang D, *et al*. Nitrite and nitrate reduction drive sediment microbial nitrogen cycling in a eutrophic lake. *Water Res.* **220**, 118637 (2022).
10. Chin KJ, Esteve-Nunez A, Leang C, Lovley DR. Direct correlation between rates of anaerobic respiration and levels of mRNA for key respiratory genes in *Geobacter sulfurreducens*. *Appl Environ. Microbiol.* **70**, 5183-5189 (2004).
11. Liu F, Rotaru AE, Shrestha PM, Malvankar NS, Nevin KP, Lovley DR. Magnetite compensates for the lack of a pilin-associated *c*-type cytochrome in extracellular electron exchange. *Environ. Microbiol.* **17**, 648-655 (2015).
12. Zhang H*, et al. Paracoccus versutus* KS293 adaptation to aerobic and anaerobic denitrification: Insights from nitrogen removal, functional gene abundance, and proteomic profiling analysis. *Bioresour. Technol.* **260**, 321-328 (2018).
13. Huang C*, et al.* Relationship between functional bacteria in a denitrification desulfurization system under autotrophic, heterotrophic, and mixotrophic conditions. *Water Res.* **188**, 116526 (2021).
14. Henry S, Bru D, Stres B, Hallet S, Philippot L. Quantitative detection of the *nosZ* gene, encoding nitrous oxide reductase, and comparison of the abundances of 16S rRNA, *narG*, *nirK*, and *nosZ* genes in soils. *Appl. Environ. Microbiol.* **72**, 5181-5189 (2006).
15. Jones CM, Graf DR, Bru D, Philippot L, Hallin S. The unaccounted yet abundant nitrous oxide-reducing microbial community: a potential nitrous oxide sink. *ISME J.* **7**, 417-426 (2013).
16. He X, et al. Evolution of corresponding resistance genes in the water of fish tanks with multiple stresses of antibiotics and heavy metals. *Water Res*. **124**, 39-48 (2017)
