# Supplementary material for: Electron shuttling promotes denitrification and mitigates nitrous oxide emissions in lakes
Source: Nat Commun. 2025 Sep 29;16:8564. doi: 10.1038/s41467-025-63601-0 (PMC12480950; doi:10.1038/s41467-025-63601-0)
Supplement: Supplementary file 3 — Reporting Summary [file 41467_2025_63601_MOESM3_ESM.pdf]

nature portfolio

Corresponding author(s): Kang Song; Min Deng; Fengchang Wu

Last updated by author(s): Aug 5, 2025

Reporting Summary

Nature Portfolio wishes to improve the reproducibility of the work that we publish. This form provides structure for consistency and transparency in reporting. For further information on Nature Portfolio policies, see our Editorial Policies and the Editorial Policy Checklist.

Statistics

For all statistical analyses, confirm that the following items are present in the figure legend, table legend, main text, or Methods section.

n/a	Confirmed	
☐	☒	The exact sample size (*n*) for each experimental group/condition, given as a discrete number and unit of measurement
☐	☒	A statement on whether measurements were taken from distinct samples or whether the same sample was measured repeatedly
☐	☒	The statistical test(s) used AND whether they are one- or two-sided<br>*Only common tests should be described solely by name; describe more complex techniques in the Methods section.*
☐	☒	A description of all covariates tested
☐	☒	A description of any assumptions or corrections, such as tests of normality and adjustment for multiple comparisons
☐	☒	A full description of the statistical parameters including central tendency (e.g. means) or other basic estimates (e.g. regression coefficient) AND variation (e.g. standard deviation) or associated estimates of uncertainty (e.g. confidence intervals)
☐	☒	For null hypothesis testing, the test statistic (e.g. *F*, *t*, *r*) with confidence intervals, effect sizes, degrees of freedom and *P* value noted<br>*Give P values as exact values whenever suitable.*
☒	☐	For Bayesian analysis, information on the choice of priors and Markov chain Monte Carlo settings
☒	☐	For hierarchical and complex designs, identification of the appropriate level for tests and full reporting of outcomes
☐	☒	Estimates of effect sizes (e.g. Cohen's *d*, Pearson's *r*), indicating how they were calculated

Our web collection on statistics for biologists contains articles on many of the points above.

Software and code

Policy information about availability of computer code

Data collection	No software was used in this study for data collection.
Data analysis	R v4.0.4; R studio v2023.09.0; GraphPad Prism v9.0.0; qiime2 v2024.5; biom-format v2.1.14; fastp v0.23.4; MEGAHIT v1.2.9; Bowtie2 v2.5.1; samtools v1.19; MetaBAT2 v2.15; dRep v3.4.0; CheckM v1.2.2; GTDB-Tk v2.4.0; CoverM v0.6.1; Prodigal v2.6.3; HMMER v3.4; bbmap v39.01; Muscle v5.1; trimAl v1.4.1; IQ-TREE v2.3.1.<br>All code used for data processing and figure generation has been deposited in the GitHub (https://doi.org/10.5281/zenodo.16416644) and figshare (https://doi.org/10.6084/m9.figshare.28050473).

For manuscripts utilizing custom algorithms or software that are central to the research but not yet described in published literature, software must be made available to editors and reviewers. We strongly encourage code deposition in a community repository (e.g. GitHub). See the Nature Portfolio guidelines for submitting code & software for further information.

Data

Policy information about availability of data

All manuscripts must include a data availability statement. This statement should provide the following information, where applicable:

- Accession codes, unique identifiers, or web links for publicly available datasets
- A description of any restrictions on data availability
- For clinical datasets or third party data, please ensure that the statement adheres to our policy

The following databases were used in this study: Kraken2DB, GTDB r220, NCBI, Pfam, KEGG, TIGRfam, Silva v138.1. The 16S rRNA gene high-throughput sequencing data generated in this study have been deposited in the NCBI database under accession code SRP550967 (https://www.ncbi.nlm.nih.gov/sra/?term=SRP550967). The metagenomic sequencing data generated in this study have been deposited in the NCBI database under accession code SRP551189 (https://www.ncbi.nlm.nih.gov/sra/?term=SRP551189). The supplementary methods, figures and tables generated in this study are provided in the Supplementary Information. The processed MAGs (https://doi.org/10.6084/m9.figshare.28013063) and representative nosZ sequences (https://doi.org/10.6084/m9.figshare.28041077) data are available at figshare. The raw figures data generated in this study have been deposited in the Github (https://doi.org/10.5281/zenodo.16416644) and figshare (https://doi.org/10.6084/m9.figshare.28050437).

Research involving human participants, their data, or biological material

Policy information about studies with human participants or human data. See also policy information about sex, gender (identity/presentation), and sexual orientation and race, ethnicity and racism.

Reporting on sex and gender	N/A
Reporting on race, ethnicity, or other socially relevant groupings	N/A
Population characteristics	N/A
Recruitment	N/A
Ethics oversight	N/A

Note that full information on the approval of the study protocol must also be provided in the manuscript.

Field-specific reporting

Please select the one below that is the best fit for your research. If you are not sure, read the appropriate sections before making your selection.

☐ Life sciences ☐ Behavioural & social sciences ☒ Ecological, evolutionary & environmental sciences

For a reference copy of the document with all sections, see nature.com/documents/nr-reporting-summary-flat.pdf

Ecological, evolutionary & environmental sciences study design

All studies must disclose on these points even when the disclosure is negative.

Study description	This study investigates how extracellular electron transfer (EET) capability influences N_2 and N_2O greenhouse gas emissions in Lake Taihu (n = 12 biological replicates). We quantified key environmental factors and functional gene abundance to characterize emission patterns. Through paired experiments with sediment samples amended with humic substances (n = 8 biological replicates), we analyzed the impacts of humic amendment on EET capacity, denitrification rates, N_2O accumulation, and functional gene abundance/expression. Microbial community composition was profiled using high-throughput sequencing, while metagenomic analysis revealed EET pathways.
Research sample	Twelve sampling sites were selected across four influent rivers influenced by urban and agricultural land use to examine sediments with varying maximum power density gradients. Field investigations and sample collections were conducted in late April 2024.
Sampling strategy	At each site, triplicate water samples and one surface sediment were collected. For dissolved N2O analysis, three water samples were collected 30 cm below the surface water using 200 mL plastic syringes fitted with a three-way stopcock. Three water samples were collected and carefully transferred into 12 mL vials at each sampling site.<br>By establishing sampling sites across different land-use and submerged plant zones, a wide range of humic substance concentrations was obtained. This range encompasses the previously reported humic substance concentrations in shallow, eutrophic lakes.
Data collection	Field investigations were conducted by Yanlin Xiao, Yuren Wang, Min Deng, Shuni Zhou, Yongxia Huang, Senbati Yeerken. Sites information, weather conditions, and water quality data were recorded using ballpoint pens on pre-printed data collection forms.

Timing and spatial scale	Field investigations and sample collections were conducted daily from April 16th to 18th, 2024. Sampling sites spanned both submerged and non-submerged vegetation zones (E120°0′31″-E120°24′19″, N31°26′34″-N31°33′68″), covering a straight-line distance of approximately 40 kilometers.
Data exclusions	No data was excluded for analyses.
Reproducibility	All experiments were independently repeated at least once to ensure reproducibility, and all attempts were successful for every experiment.
Randomization	All samples were selected to analyze the functional group characteristics of HS under varying MPD conditions. Eight biological replicates were used for incubation experiments, comprising three L-MPD samples (WX_2, WX_3, WY_1) and all M-MPD and H-MPD samples. The three L-MPD samples were randomly selected. DX_1, LX_3, and LX_1 were randomly selected as representative L-MPD, M-MPD, and H-MPD samples for XPS analysis.
Blinding	No formal blinding was applied in this study, consistent with its design. No predefined groups guided sampling; samples were collected based on objective environmental conditions, making group concealment irrelevant. Initial analyses used the full dataset without prior stratification. Significant differences in "L-MPD samples" emerged from post-hoc exploratory analysis of objective data, with no predefined groups to introduce bias. For the humic substance addition indoor experiment, cultivation bottles from both groups were randomly arranged in the incubator, ensuring that researchers were unaware of group identities during daily maintenance and initial data recording.

Did the study involve field work? [x] Yes [ ] No

Field work, collection and transport

Field conditions	Field investigations and sediment sampling were performed during late April 2024, exclusively on sunny days. Measured air temperatures ranged from 21.3 to 21.5°C during sampling operations.
Location	The sampling sites spanned both submerged and non-submerged vegetation zones (E120°0′31″-E120°24′19″, N31°26′34″-N31°33′68″).
Access & import/export	N/A
Disturbance	The sampling region has long practiced a summer rice-winter wheat double-crop rotation system. Field investigations and sample collections were conducted in late April 2024, coinciding with the wheat maturity stage. During this phase, farmers typically avoid large-scale nitrogen fertilization due to diminished crop root absorption capacity and risks of delayed maturity. While rainfall is a primary driver of nitrogen loss from farmland, April is a dry season with minimal precipitation in the region. Sampling was conducted on sunny days to avoid short-term drastic changes in nitrogen nutrient concentrations, with $|Z\text{-score}| < 3$ confirming no statistical outliers.

Reporting for specific materials, systems and methods

We require information from authors about some types of materials, experimental systems and methods used in many studies. Here, indicate whether each material, system or method listed is relevant to your study. If you are not sure if a list item applies to your research, read the appropriate section before selecting a response.

Materials & experimental systems

n/a	Involved in the study	
[x]	[ ]	Antibodies
[x]	[ ]	Eukaryotic cell lines
[x]	[ ]	Palaeontology and archaeology
[x]	[ ]	Animals and other organisms
[x]	[ ]	Clinical data
[x]	[ ]	Dual use research of concern
[x]	[ ]	Plants

Methods

n/a	Involved in the study	
[x]	[ ]	ChIP-seq
[x]	[ ]	Flow cytometry
[x]	[ ]	MRI-based neuroimaging

Plants

Seed stocks	N/A
Novel plant genotypes	N/A
Authentication	N/A
